# TB and HIV induced immunosenescence: where do vaccines play a role?

**DOI:** 10.3389/fragi.2024.1385963

**Published:** 2024-06-05

**Authors:** Mona Singh, Bhumika Patel, Michael Seo, Phillip Ahn, Nejma Wais, Haley Shen, SriHarsha Nakka, Priya Kishore, Vishwanath Venketaraman

**Affiliations:** ^1^ College of Osteopathic Medicine of the Pacific, Western University of Health Sciences, Pomona, CA, United States; ^2^ Kempegowda Institute of Medical Sciences, Bengaluru, Karnataka, India; ^3^ Masters of Public Health, Chamberlain University, Addison, IL, United States

**Keywords:** immunosenescence, *M. tuberculosis*, Vaccine, BCG, HIV

## Abstract

This paper tackles the complex interplay between Human Immunodeficiency virus (HIV-1) and *Mycobacterium tuberculosis* (*M*. *tuberculosis*) infections, particularly their contribution to immunosenescence, the age-related decline in immune function. Using the current literature, we discuss the immunological mechanisms behind TB and HIV-induced immunosenescence and critically evaluate the BCG (*Bacillus* Calmette-Guérin) vaccine’s role. Both HIV-1 and *M. tuberculosis* demonstrably accelerate immunosenescence: *M. tuberculosis* through DNA modification and heightened inflammation, and HIV-1 through chronic immune activation and T cell production compromise. HIV-1 and *M. tuberculosis* co-infection further hastens immunosenescence by affecting T cell differentiation, underscoring the need for prevention and treatment. Furthermore, the use of the BCG tuberculosis vaccine is contraindicated in patients who are HIV positive and there is a lack of investigation regarding the use of this vaccine in patients who develop HIV co-infection with possible immunosenescence. As HIV does not currently have a vaccine, we focus our review more so on the BCG vaccine response as a result of immunosenescence. We found that there are overall limitations with the BCG vaccine, one of which is that it cannot necessarily prevent re-occurrence of infection due to effects of immunosenescence or protect the elderly due to this reason. Overall, there is conflicting evidence to show the vaccine’s usage due to factors involving its production and administration. Further research into developing a vaccine for HIV and improving the BCG vaccine is warranted to expand scientific understanding for public health and beyond.

## 1 Introduction

The Human Immunodeficiency virus type 1 (HIV-1) leads to the destruction of CD4^+^ T cells which leads to the eventual failure of the host immune system. HIV can enter these target cells by binding primarily to chemokine co-receptor 5 (CCR5), CC-chemokine receptor 4 (CXCR4), and target receptors/co-receptors CD4. The CCR5-tropic HIV dominates during the onset of infection, while the CXCR4 HIV dominates during the chronic phase. HIV can evade the immune system using several mechanisms. It facilitates the destruction of CD4^+^ T cells by chronic immune activation, accelerated destruction, and impairing the regeneration of new T cells from precursor cells (Shankar et al., 2014).

*Mycobacterium tuberculosis* (*M. tuberculosis*) is a complex organism primarily affecting the respiratory system, with the potential for dissemination in immunocompromised individuals (Luca and Mihaescu, 2013). The most common route of *M. tuberculosis* infection is through inhaling aerosolized bacilli into the respiratory tract. Cells like alveolar macrophages and Langerhans cells in the airway mucosa capture these bacilli and transport them to nearby lymph nodes, initiating immune responses. However, *M. tuberculosis* prevents the fusion of the phagosome, where the bacilli are enclosed, with the lysosome, allowing it to multiply within this vesicle (Luca and Mihaescu, 2013). While the exact mechanism of evasion from lysosomal fusion is unclear, it may involve structures like the arabinogalactan mycolate lipoarabinomannan (LAM) complex enriched in acid-fast cell-wall structures known as Wax-D (Luca and Mihaescu, 2013). Wax-D likely modifies the phagosome membrane, hindering normal fusion with lysosomes (Luca and Mihaescu, 2013). The bacteria’s survival is also supported by the formation of granulomas, collections of various immune cells such as macrophages, fibroblasts, lymphocytes, dendritic cells, and neutrophils, forming granulomas rich in multinucleated giant cells that attempt to contain the infection (Luca and Mihaescu, 2013). These granulomas aim to restrict the replication of the bacilli. Despite this defense, *M. tuberculosis* persists within the granuloma, and any disruption to its integrity ultimately leads to the reactivation of latent tuberculosis infection. This reactivation results in the dissemination of the bacteria through the lymphatic and hematogenous systems, characterized by specific lesions and necrosis particularly in areas of the lungs with reduced air circulation and lymphatic drainage, leading to the development of severe active tuberculosis (Luca and Mihaescu, 2013). This is characterized by the formation of a Ghon focus, particularly involving the hilar lymph nodes, and the occurrence of caseous necrosis, commonly in the apical segment of the lungs due to inefficient air pumping mechanisms and lymphatic distribution (Luca and Mihaescu, 2013).

*Mycobacterium tuberculosis* and HIV are known to induce immunosenescence, a process observed in elderly individuals (Luca and Mihaescu, 2013). Immunosenescence refers to the gradual decline of the body’s immune system, resulting in weakened responses to vaccinations and infections. It involves alterations and weakening of T-cell subsets and often leads to the shrinking of lymphoid organs, ultimately causing a decline in both T- and B-cell functions (Shankar et al., 2014; Shankar et al., 2015). Recent research indicates that immunosenescence can affect both adaptive and innate immune cells, with T-cells being particularly susceptible (Shankar et al., 2015). This can result in an unstable response to antigens, reduced levels of CD4^+^ or CD8^+^ T cells, decreased numbers of naïve cells, and an accumulation of memory T cells, including CD57^+^ cells, which are associated with senescence (Sauce et al., 2009). CD57 serves as a marker for senescence and is linked to replicative senescence in both CD4^+^ and CD8^+^ T cells in older adults, as well as in persistent viral infections (Shankar et al., 2015). Both HIV and *M. tuberculosis* have been shown to promote the expansion of CD57^+^CD8^+^ T cells, contributing to the immunopathogenesis of each disease. *Mycobacterium tuberculosis* leads to an increase in specific immune markers such as CD57^+^ and CD8^+^ T cells, indicating its involvement in infection-induced immunosenescence (Shankar et al., 2015). Furthermore, the expansion of CD57^+^CD8^+^ T cells in response to *M. tuberculosis* infection exhibits heightened cytokine and cytolytic potential, characterized by the secretion of TNF-α and IL-6 (Shankar et al., 2015). This dysregulation may eventually exacerbate HIV progression in co-infected individuals.

HIV-1 and *M. tuberculosis* infections induce immunosenescence. There are currently studies showing the interaction between HIV and *M. tuberculosis* co-infection and their role in immunosenescence (Shankar et al., 2014). Although the exact mechanism is still unclear, it has been shown that both TB and HIV exert influence on the hosts’ immune system (Shankar et al., 2014; Shankar et al., 2015). Several *in vitro* studies have demonstrated that phagocytosis of *M. tuberculosis* induces macrophage activation, and production of proinflammatory cytokines, namely, TNF-α, IL-1β and IL-6 by macrophages, which is also known to enhance replication of HIV-1 (Shankar et al., 2014). The growth of *M. tuberculosis* was found to be notably higher in monocyte-derived macrophages (MDMs) infected with HIV-1 compared to HIV-1-negative cells (Shankar et al., 2014). Additionally, the introduction of TNF-α to HIV-1-infected MDMs led to a significant increase in *M. tuberculosis* growth, whereas TNF-α had no impact on *M. tuberculosis* growth in uninfected MDMs. Furthermore, in latently HIV-1-infected promonocytic cells, the phagocytosis of *M. tuberculosis* triggered viral production, while in acutely HIV-1-infected MDMs, there was an increase in *M. tuberculosis* growth. Moreover, co-infection with HIV-1 resulted in a higher bacilli burden in cell cultures and accelerated growth of *M. tuberculosis*, subsequently promoting HIV-1 replication (Shankar et al., 2014). The co-infection of HIV-1/*M. tuberculosis* also synergistically reduced macrophage viability, elevated levels of proinflammatory cytokines, and this effect appeared specific to *M. tuberculosis* rather than other mycobacteria species (Shankar et al., 2014). This shows that there is indeed a co-infection between HIV1 and *M. tuberculosis* and that they trigger a complex immune response which over time becomes exhausted, leading to immunosenescence. Therefore, investigating the exact influence that a *M. tuberculosis*/HIV co-infection can have on immunosenescence and the failure of the immune system is of utmost importance.

Overall, it is critical to understand how vaccine responses are affected due to infection induced immunosenescence. The BCG vaccine is used against TB disease (Norouzi et al., 2012). Many foreign-born persons have received this vaccine, and it is used in many countries with a high prevalence of TB such as in Sub Saharan Africa, Eastern Europe, and Asia. However, since the BCG vaccine is a live vaccine, it is contraindicated in individuals with immunodeficiency diseases (Norouzi et al., 2012). Immunocompromised hosts may be vulnerable not only to mycobacterial diseases, but also other lethal infections and complications that may come along with the BCG vaccine. Due to immunosenescence from *M. tuberculosis* and HIV co-infection, the response by the *Bacillus* Calmette-Guérin (BCG) vaccine can potentially be altered, although further research needs to be done on this topic. Although the vaccine has shown benefit for the younger population, the effectiveness of the vaccine as a whole is controversial, and thus has further limitations for its use, as we discuss in further detail throughout the paper.

## 2 Pathogenesis and culprits of TB induced immunosenescence

### 2.1 CD8^+^ T cells in immunosenescence

**FIGURE 1:**
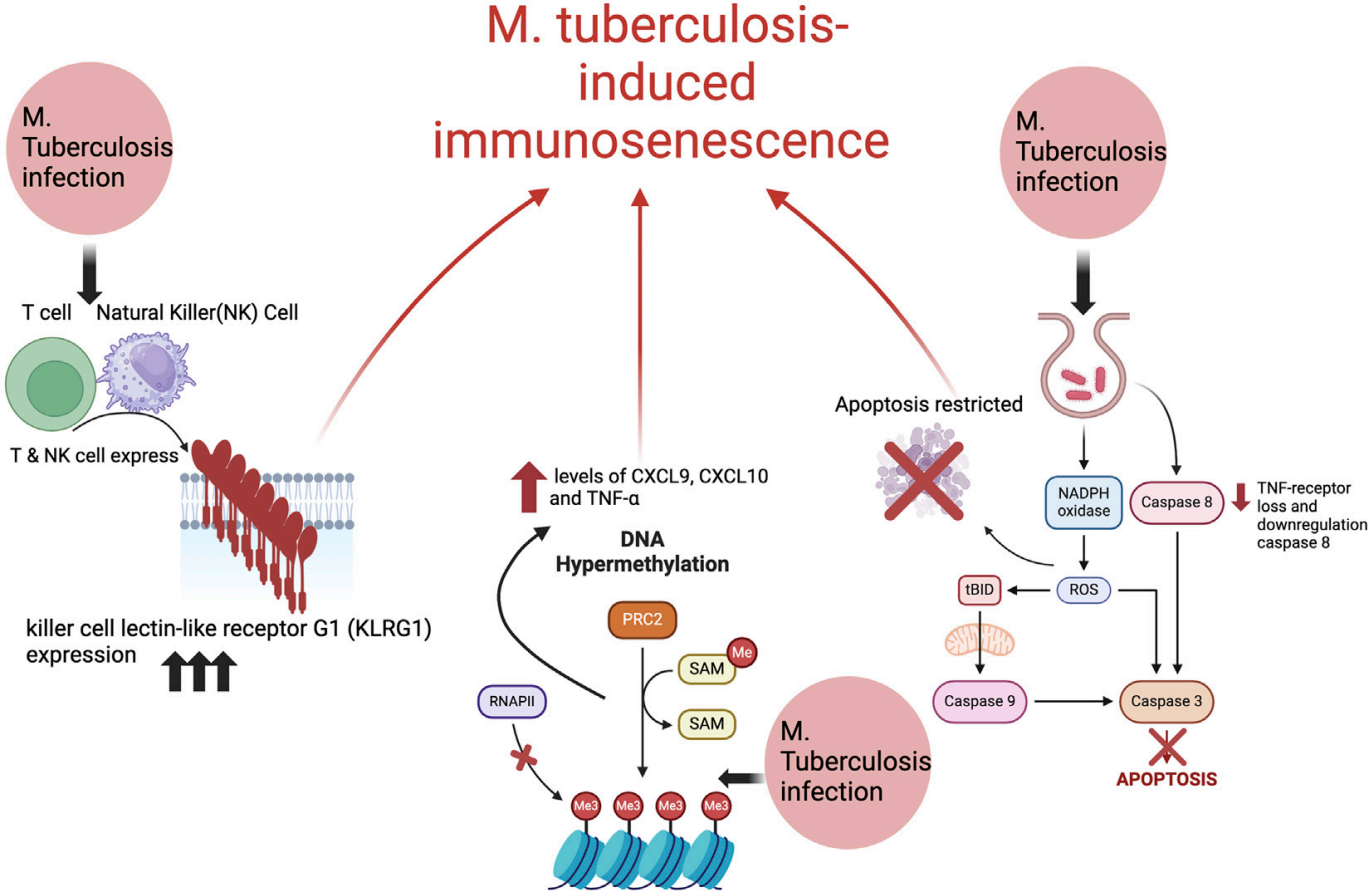
The possible mechanisms of *Mycobacterium tuberculosis* induced immunosenescence. Created with BioRender.com.

*Mycobacterium tuberculosis* induced immunosenescence is mediated by changes in gene expression, DNA methylation and hypermethylation, as well as increased reactive oxygen species (ROS) and pro-inflammatory cytokines (Reyes et al., 2023) (Figure 1). There are studies conducted in guinea pigs and humans which have revealed that *M. tuberculosis* leads to alterations in gene expression and DNA methylation, including hypermethylation (Bobak et al., 2022). This is linked to oxidative stress-induced senescence and elevated levels of the senescence associated proteins like CXCL9, CXCL10, and TNF-α (Bobak et al., 2022), which will further be discussed in this section. Additionally, a study in older mice revealed that the presence of aged memory CD8^+^ T cells correlated with decreased control of *M. tuberculosis* (Reyes et al., 2023). These alterations by *M. tuberculosis* directly induce immunosenescence and the aging of the immune system such as the CD8^+^ T cells. Other culprits that will be discussed is the senescence marker killer cell lectin-like receptor G1 (KLRG1), and other inflammatory markers involved like interferons and tumor necrosis factor, interleukin (TNF) 1.

### 2.2 CD4^+^ T cells in immunosenescence

Expression of specific T cell differentiation markers have been known to increase during *M. tuberculosis* infection, notably the senescence marker killer cell lectin-like receptor G1 (KLRG1), which is expressed by natural killer and differentiated T cells (Cyktor et al., 2013; Reyes et al., 2023). It was found that KLRG1 expression on T cells increases during *M. tuberculosis* infection during aging and decreases after treatment of TB, suggesting an association with KLRG1 expression and disease progression (Cyktor et al., 2013; Reyes et al., 2023). Comparing wild-type mice with KLRG1^−/−^ mice, the latter had a significantly longer survival, beyond 600 days, and maintained lower levels of *M. tuberculosis* throughout chronic infection from day 60 onward (Cyktor et al., 2013). The knockout mice had significantly enhanced CD4^+^ T cell responses to *M. tuberculosis*, suggesting that those with KLRG1 had diminished immune function (Cyktor et al., 2013). Thus, TB is associated with diminished CD4^+^ T cell response through KLRG1, with KLRG1 being a marker involved in the immunosenescence of *M. tuberculosis*.

### 2.3 DNA methylation

Comparing humans and animal models with TB, there is a significant overlap in DNA hypermethylation changes in CD4^+^ and CD8^+^ T cells, demonstrating the role of *M. tuberculosis* in epigenetic modifications in genes related to immune system cells that decrease their function over time (Reyes et al., 2023). Studies conducted in guinea pigs and humans showed that *M. tuberculosis* induces changes in gene expression, DNA methylation and hypermethylation. This is associated with oxidative stress-induced senescence with increased levels of senescence associated proteins CXCL9, CXCL10 and TNF-α (Bobak et al., 2022). Particularly, these investigations noted similar epigenetic changes in transcription factors like NFKBIA, TCF7, CIITA, MYC, NFAT, and DNMT1/3A in both guinea pigs and aging humans infected with *M. tuberculosis* (Bobak et al., 2022). DNA methylation has previously been associated with aging; thus, *M. tuberculosis*’ influence on hypermethylation advances this part of aging significantly (Reyes et al., 2023). In studies done on guinea pigs and humans, it demonstrated that DNA hypermethylation is associated with decreased immune responsiveness, and hypermethylation did not return to normal when measured 6 months after completion of successful TB medical therapy, too (Bobak et al., 2022; Reyes et al., 2023). Another study compared biological age against chronological age using the Horvath clock, which calculates biological age based on DNA methylation (Bobak et al., 2022). Application of the Horvath clock to TB patients demonstrated an average increase in DNA methylation age by 12.7 years older than the patients’ chronological ages (Bobak et al., 2022). This increase in epigenetic age was measured for at least 12 months from TB diagnosis, which was also 6 months after completion of therapy (Bobak et al., 2022). These findings do not exclude the possibility that individuals with increased DNA methylation to begin with are at increased risk of TB; however, the association is strong. DNA methylation has been shown to be positively associated with all-cause mortality (Olmo-Fontánez and Turner, 2022). Due to the immunosenescence through DNA hypermethylation, this may lead to poorer responses to vaccines.

### 2.4 Inflammation and inflammatory markers

While age itself is a risk factor for increased inflammation, the inflammation in TB pathogenesis is further caused by impressive survival mechanisms of *M. tuberculosis*. Pro-inflammatory cytokines, such as interferons, tumor necrosis factor, interleukin (TNF) 1, microRNAs, and eicosanoids interact during *M. tuberculosis* infection (Olmo-Fontánez and Turner, 2022). Recent investigations show neutrophils’ involvement with oxidase-dependent anti-mycobacterial properties, increasing ROS interactions with local tissues (Kaufmann and Dorhoi, 2013). Moreover, epithelial cells surrounding infected phagocytes release matrix metalloproteinase-9 (MMP), facilitating local inflammation, and MMP-1 produced by infected macrophages, which facilitates lung destruction and granuloma formation. In response to *M. tuberculosis* infection, the immune system attempts to induce apoptosis, but several studies suggest that *M. tuberculosis* has developed mechanisms to restrict apoptosis and thus increase the inflammatory reaction of host cells (Kaufmann and Dorhoi, 2013). During tuberculosis infection, anti-inflammatory lipoxin A synthesis lowers prostaglandin E2 abundance, and thus, inhibits apoptotic envelope and plasma repair mechanisms, causing necrosis rather than apoptosis (Kaufmann and Dorhoi, 2013). As cell membranes deteriorate, metabolites of arachidonic acid act as second messengers for TNF-alpha activation, a cytokine that can initiate apoptosis through the extrinsic pathway (Kaufmann and Dorhoi, 2013). To counteract, *M. tuberculosis* induces TNF-receptor loss and downregulates caspase-8, which is involved in the extrinsic pathway (Kaufmann and Dorhoi, 2013) (Figure 1). By directly increasing pro-inflammatory cytokines and diverting the host cell mechanisms for apoptosis, inflammation is increased in the host and can facilitate immunosenescence.

In summary, *M. tuberculosis* immunosenescence is facilitated by gene expression changes, such as involvement of KLRG1, DNA hypermethylation which is associated with aging and further facilitated by tuberculosis infection, and inflammation and restriction of apoptosis that causes chronic damage to host tissue over time. This exhaustion of the immune system may significantly affect vaccine response as a result.

## 3 Pathogenesis and culprits of HIV induced immunosenescence

Acquired Immunodeficiency Syndrome (AIDS), once considered a fatal diagnosis, has now evolved into a chronic disease with the advent of antiretroviral therapy (ART). The life expectancy of patients infected with HIV has significantly improved from a few years to several decades (Trickey et al., 2022). However, even with the advent of ART, patients undergoing ART still die earlier than those without HIV infection and suffer from numerous health sequelae involving multiple organ systems (Deeks et al., 2012). Such health conditions are usually only seen later in life as the result of immunosenescence (Stahl and Brown, 2015). Thus, immunosenescence as a result of HIV infection can be fatal even among those with ART. The prematurity and increase in traditionally age-related mortality observed in HIV-infected patients requires further research into HIV-induced immunosenescence. Persistent immune activation as well as thymic involution will be discussed as possible culprits of HIV-induced immunosenescence.

**FIGURE 2:**
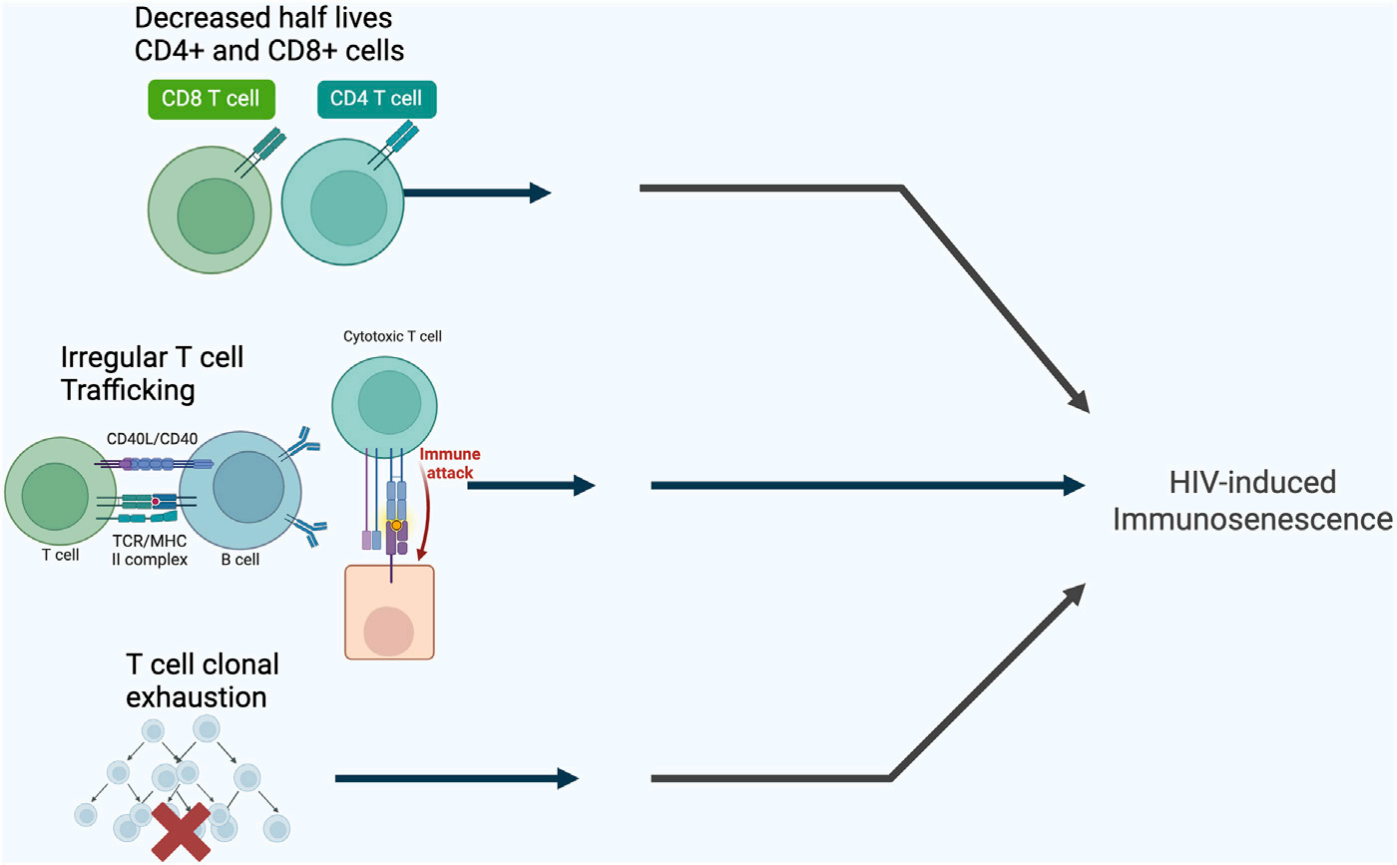
The possible mechanisms of HIV-induced immunosenescence. Created with BioRender.com.

The persistent immune activation seen in HIV-infected patients results in the exhaustion of T-cell and memory T-cell pools that is measured by a decrease in the half-lives of CD4^+^ and CD8^+^ T cells, irregular T-cell trafficking within T-cell subsets, and selective T-cell clonal exhaustion (Papagno et al., 2004; Appay et al., 2007; Sokoya et al., 2017) (Figure 2). This directly contrasts conventional beliefs that the main culprit of reduced CD4^+^ count in HIV-infected patients is due to apoptosis of host cells during HIV replication, and instead offers immune activation as the larger contributor in T-cell depletion seen in HIV-infected patients. Silvestri et al. (2003) conducted seminal research on nonpathogenic simian immunodeficiency virus (SIV) infection of sooty mangabeys, the natural host of SIV, demonstrating the lack of correlation between CD4^+^ T cell counts and SIV-plasma viremia, positing that SIV viral replication alone cannot account for the progressive depletion of CD4^+^ T cells leading to AIDS. The progressive decline in the immune system is not only due to viral replication, but also T cell activation. Hunt et al. (2008) also demonstrates in their study that elite HIV controllers—those able to maintain undetectable plasma HIV RNA levels (below 75 copies/mL) without the use of ART—have higher T cell activation levels than HIV negative patients, and demonstrates that lower CD4^+^ T cell counts were associated with higher levels of activated CD4^+^ T cells and CD8^+^ T cells in these controllers, also suggesting that the persistent immune activation associated with chronic HIV infection is likely less related to the viral load itself, but rather associated with T cell activation. This demonstrates that HIV induced immunosenescence consists of the progressive immune activation involving viral replication and the activation of T cells.

An additional culprit of HIV induced immunosenescence is thymic involution. The natural, age-related involution of the thymus and consequent reduction of thymopoiesis precedes T-cell related immune-incompetence in older individuals and is a key player in the immunosenescence observed in the older population (Gui et al., 2012). The thymus is an early site for HIV-1 infection: CD4 is expressed not only on mature (CD3^+^CD4^+^CD8^−^) T cells, but also on less mature (CD3−/+ CD4^+^CD8^+^) thymocytes and intrathymic T progenitor (ITTP, CD3^−^CD4^+^CD8^−^) cells (Meissner et al., 2003). Due to CD4 expression on nearly all thymocytes, the extent of thymic HIV-1 infection and subsequent thymic destruction is determined primarily by co-receptor tropism: Studies using SCID-hu Thy-Liv mice and human thymocyte tissue cultures show X4-tropic (CXCR4) HIV-1 strains result in rapid destruction of all thymocyte subpopulations, while R5-tropic (CCR5) HIV-1 strains result in slower destruction of stromal cells and mature thymocytes (Ye et al., 2004). As such, HIV infection, akin to the natural aging process in the elderly, induces thymic involution and subsequent immunosenescence.

## 4 TB and HIV induced immunosenescence

Current studies on HIV/*M. tuberculosis* co-infections have provided insight into how these two pathogens work synergistically to hinder the host immune response and cause premature immunosenescence (Bruchfeld et al., 2015). It has been widely recognized that TB and HIV both exert a significant influence on the host immune system (Appay et al., 2007; Diedrich and Flynn, 2011; Bruchfeld et al., 2015). However, there is an urgent need to elucidate the mechanism by which the immune system is compromised because of the interaction between the two pathogens.

HIV-*M. tuberculosis* coinfection appears to play a role in modifying the process of cellular differentiation in naïve T cells. Upon antigenic activation, naïve T cells undergo differentiation to develop a more unique phenotype and to become more specialized in their function. T cells divide into various subsets that each carry out a distinct function. In a study investigating phenotypic variability of T cells during different viral infections, three distinct subcategories were established based on the differential expression of costimulatory molecules CD27 and CD28 during the early, intermediate, and late stages of CD8^+^ T cell proliferation (Appay et al., 2002; Kovaiou et al., 2005). Another study found that CD27 and CD28 can also be used to determine the stage of T-cell activation and proliferation (Kushner et al., 2010; Diedrich and Flynn, 2011). Lower expressions of CD28 were found to be indicative of later stages of T cell differentiation and proliferation. It also indicates that T cells may have entered a state of immunosenescence which is characterized by shorter telomeres and a decreased ability to replicate (Choremi-Papadopoulou et al., 1994). CD27 was found to have modulatory effects on T-cell functions and demonstrated a stronger correlation with the proliferative potential of T cells. Additionally, it has been established that patients with persistent HIV infections are found to have lower expressions of CD27 and CD28 (Hamann et al., 1999; Lee et al., 2014). This reflects advanced stages of T cell differentiation and a greater number of proliferation cycles that have occurred because of the host immune response to pathogens. Therefore, HIV-*M. tuberculosis* co-infection appears to accelerate the rate of downregulating CD27 and CD28 when compared to an HIV infection alone.

Research also shows that chronic HIV infection may inhibit the maturation of CD8^+^ T cell subsets leading to an excess number of cells presenting in the intermediate differentiation stage (Mojumdar et al., 2012; Walker et al., 2013; Lee et al., 2014). This leads to a decline in the efficacy of cytotoxic and cytokine responses when immune cells are presented with antigens. Despite the inhibitory effect that HIV infection has on T-cell differentiation, HIV-TB co-infection presents with an accelerated decline in CD27 and CD28 (Mojumdar et al., 2012; Walker et al., 2013; Lee et al., 2014). Hence it is speculated that T-cell differentiation is expedited in HIV- *M. tuberculosis* co-infection due to the predominant effects that *M. tuberculosis* exerts on the host immune response.

Despite global efforts to reduce the impact of TB over recent decades, morbidity due to TB has continued to escalate (Walker et al., 2013). This increase has been strongly correlated with the emergence of the HIV-1 epidemic, and HIV-1 is currently recognized as the greatest risk factor for TB acquisition and disseminated TB infections. In a 2012 study assessing TB incidence rates during 8 years of follow up of antiretroviral therapy (ART), it was demonstrated that TB rates continued to be significantly higher in HIV-infected individuals despite receiving ART and restoring CD4 cell counts (Walker et al., 2013). Even during the first few years immediately after acquiring HIV, the risk of TB acquisition and disseminated TB was found to be substantially elevated (Gupta et al., 2012).

It is becoming increasingly clear that chronic HIV infection accelerates the onset of chronic immune activation (CIA) that subsequently leads to premature immunosenescence, thus exacerbating the risk of *M. tuberculosis* infection (Hazenberg et al., 2003; Papagno et al., 2004; Rodriguez et al., 2006; Effros, 2007; Gonzalez et al., 2009; Dock and Effros, 2011). However, recent studies also suggest that *M. tuberculosis* may have an equally detrimental effect on HIV disease by enhancing viral transmission and facilitating the entry of HIV into immune cells (Diedrich and Flynn, 2011; Kwan and Ernst, 2011; Shankar et al., 2014). It has been speculated that *M. tuberculosis* infection impacts the host immune response by allowing HIV to overcome anti-viral responses and to undergo rapid amplification by the formation of granulomas. Hence, it is possible to correlate TB mono-infection with the earlier onset of AIDS and *vice versa*. Based on the effects that *M. tuberculosis* and HIV infection exert on each other and on the host immune system, there is a strong impetus to further investigate the mechanism behind how CIA in HIV- *M. tuberculosis* coinfection leads to the earlier onset of immunosenescence.

## 5 Discussion of the BCG vaccine

### 5.1 History of the BCG vaccine and its efficacy

In 1921, the Bacille Calmette-Guerin (BCG) vaccine was administered orally to an infant whose mother had died of tuberculosis a few hours after giving birth. At the time, this method of vaccination was deemed to be safe and effective, until the Lübeck disaster occurred. In 1930, there was a plan to vaccinate newborn babies at the Lübeck General Hospital in Germany. However, after four to 6 weeks, out of 250 vaccinated, there were 73 deaths in the first year and 135 infected (Luca and Mihaescu, 2013). This incident deeply affected the confidence in the BCG vaccine. Even though there has been considerable research and development of the vaccine since then, the efficacy of the BCG vaccine against TB remains controversial. Currently, the U.S. does not officially recommend the vaccine, while 180 other countries do (Zwerling et al., 2011). Most of the countries that officially recommended the BCG vaccine have higher incidences of TB, suggesting that benefits of the vaccine outweigh its negative effects in countries where TB is more prevalent.

Today, there are many BCG vaccines in use, differing in the manner of production. Most of the BCG vaccines are attenuated, live culture preparations derived from *Mycobacterium bovis*. They are administered intradermally (Dockrell and Smith, 2017). One example is the TICE strain, which was developed at the University of Illinois from a strain originating at the Pasteur Institute. This strain includes a BCG organism that is grown in a medium composed of glycerin, asparagine, citric acid, potassium phosphate, magnesium sulfate, iron ammonium citrate, and lactose (Sharp and Dohme, 2023). Currently, neonatal BCG provides good protection against both pulmonary and disseminated tuberculosis in young children but has variable efficacy in adults when given later in life, which could be due to *M. tuberculosis* and HIV infection induced immunosenescence.

### 5.2 BCG vaccine on immunosenescence of TB and HIV

It was previously thought that the BCG vaccination increased the production of innate and adaptive cytokines upon microbial stimulation. However, it was recently found that the BCG vaccine lowers systemic inflammation in elderly people (Sharp and Dohme, 2023). Although it remains to be proven, these results suggest that the BCG vaccine could act to inhibit markers of inflammation in infectious diseases associated with immunosenescence. Another landmark study found that intravenous (IV) BCG vaccination can confer sterilizing immunity to *M. tuberculosis* in 75% of SIV positive macaques (Larson et al., 2023). This study showed there were no signs of disseminated BCG disease following BCG vaccination of SIV+ macaques (Larson et al., 2023). This is monumental, considering that one in three people living with HIV die of TB. Potential research on humans to study this further may be of benefit. Generally, the use of IV BCG administration shows promising results for clinical usage (Qu et al., 2021). Since there is not currently a vaccine for HIV in humans, further research on this topic could be life changing.

With regards to the use of BCG vaccine in co-infection of HIV and *M. tuberculosis*, the current literature is conflicting. It is not certain whether the BCG vaccination confers protection *with M. tuberculosis* and HIV co-infection. One study suggests that BCG has a modest protective effect against all forms of TB independent of HIV status, and BCG confers protection against extrapulmonary TB among HIV-negative individuals (Arbeláez et al., 2000). Another study suggests HIV infection seems to repudiate the protective effect of BCG against extrapulmonary TB (Arbeláez et al., 2000). However, another article shows that BCG vaccine may protect against active TB irrespective of HIV status in cases of Tanzania (Faurholt-Jepsen et al., 2013). Therefore, it is unclear whether the BCG vaccine is beneficial for those infected with HIV and TB.

Due to immunosenescence of HIV and *M. tuberculosis*, is not clear if the BCG vaccine has a poor response for protection. Current literature discusses the impact of BCG on T cell responses and the humoral response system. For example, Vordermeier et al. showed that mice lacking B cells were more vulnerable to TB, but notably, their specific T cell responses and the protective effects of BCG vaccination were not compromised (Tanner et al., 2019). Bosio et al. similarly found that B cell-deficient mice had similar bacterial levels in the lungs after a low-dose infection with an *M. tuberculosis* clinical strain, but also experienced less severe tissue damage and delayed bacterial spread (Tanner et al., 2019). Subsequent research demonstrated that transferring B cells into B cell knock-out (KO) mice reversed the increased susceptibility to infection and associated immunopathology (Tanner et al., 2019). However, other studies have indicated no significant role for B cells in these models, possibly due to variations in genetic backgrounds, *M. tuberculosis* strains, or infection doses (Tanner et al., 2019). There are also suggestions that the B cell compartment might contribute to disease progression (Tanner et al., 2019). The presence of abundant antibodies in the sera of patients with active TB implies B cells’ involvement in immunopathology (Tanner et al., 2019). However, the exact causal relationship remains unclear. Research on the B cell response to BCG vaccination is limited, and further study needs to be conducted on the topic (Tanner et al., 2019). Overall, those with the BCG vaccine have still experienced the exhaustion of the immune system with B cell involvement, but whether or not it is protective warrants further investigation.

## 6 Challenges regarding the BCG vaccine and future prospects

*Mycobacterium tuberculosis* is a challenging intracellular pathogen that has an extreme balance of physics for immune combat and can coexist with the infected hosts for centuries to come (Chai et al., 2020). TB vaccines should have the capability to mend moderate complex signs induced by *M. tuberculosis*, to evolve a sensitive balance between inflammation and regulatory immune response, and conserve stronghold immune responses for a long time. The BCG immunization goal must be periodically enhanced to ensure the long-term effect of the *M. tuberculosis* control measures globally.

Therefore, either shaping the vaccine route or relying on the prime-boost immune plan is the key to upscaling the immune effect of BCG. However, there are major restraints in performing the process of clinical trials. One hurdle in this evolution is the lack of accuracy and reliability of immune targets. It is difficult to overcome the Th1 immune response before the classic and reliable immune markers that are to be proven, which may lead to ignorance of the true effective immune response and enable *M. tuberculosis* to perform an immune evasion. This might prove that the sample size should be as enormous as possible and the real-time immunization evaluation should be stretched to a point when conducting TB vaccine research.

Additionally, vaccination strategies have typically focused on benefiting children, leaving older adults (over 60 years old) as a vulnerable population lacking effective vaccination approaches (Crooke et al., 2019). This is particularly concerning for individuals who have experienced immunosenescence due to conditions like TB infection or HIV, as immunosenescence not only increases their susceptibility to infections but also hampers their ability to mount protective immune responses post-vaccination (Crooke et al., 2019). The limited efficacy of vaccines, including the BCG vaccine, in individuals with immunosenescence underscores the need for improved vaccination approaches tailored to target senescence markers in older individuals, thereby enhancing vaccine effectiveness.

Several human epidemiological studies, along with a significant randomized controlled trial conducted in Brazil, have previously suggested that administering BCG re-vaccination after neonatal vaccination does not provide additional protection, with the Brazilian trial also supporting this conclusion (Tanner et al., 2019). However, a recent Phase II trial aimed at preventing *M. tuberculosis* infection in South African adolescents revealed that while BCG re-vaccination did not show efficacy in preventing initial infection (defined by a Quantiferon QFT conversion with an IFN-γ level of ≥0.35 IU per ml after day 84), it did lead to significantly lower rates of sustained QFT conversion (defined as three consecutive positive QFT results after day 84) (Tanner et al., 2019). The authors propose that although the innate immune response does not prevent initial infection, it does transport antigens to the draining lymph nodes, initiating adaptive immunity (as seen in initial QFT conversion), followed by improved bacterial control or clearance in protected individuals (Tanner et al., 2019). The discrepancies in outcomes between this trial and earlier BCG re-vaccination studies may stem from varying enrollment criteria; the recent study excluded adolescents who were not QFT-negative at baseline, and BCG’s effectiveness is believed to be highest in individuals without prior mycobacterial exposure (Tanner et al., 2019). These findings have reignited interest in the potential benefits of BCG re-vaccination, although the exact contributions of cellular and/or humoral immunity to this protection remain unclear.

Furthermore, since a vaccine for AIDS/HIV does not exist, it is important that its development includes the implication of immunosenescence, especially considering that many live with HIV/AIDS for a long time period. One study created a replica of the vaccine and how it could be used. A live attenuated simian human immunodeficiency virus (SHIV) genetically engineered to express the adjuvant molecule Ag85B (called SHIV-Ag85B) was created (Okamura et al., 2021). In cynomolgus macaques injected with SHIV-Ag85B, the virus was undetectable after 4 weeks, and robust SHIV-specific T cell responses were observed (Okamura et al., 2021). When these macaques were later challenged with pathogenic SHIV89.6P at 37 weeks post-SHIV-Ag85B injection, SHIV89.6P was not detectable after the challenge (Okamura et al., 2021). Eradication of SHIV89.6P was confirmed through adoptive transfer experiments and CD8-depletion studies. The macaques inoculated with SHIV-Ag85B also showed increased Gag-specific monofunctional and polyfunctional CD8^+^ T cells during the acute phase of the pathogenic SHIV challenge (Okamura et al., 2021). These findings suggest that SHIV-Ag85B induced strong immune responses against pathogenic SHIV, potentially paving the way for an AIDS virus infection vaccine. Looking into prospects, tailoring the vaccine to increase its response despite immunosenescence would be ultimately beneficial for its long-term efficacy for populations that need it most.

## 7 Conclusion

*Mycobacterium tuberculosis*-induced immunosenescence is orchestrated by alterations in gene expression, DNA methylation, hypermethylation, augmented ROS, and pro-inflammatory cytokines. Additionally, the persistence of immune activation alongside thymic involution emerges as potential instigators of HIV-induced immunosenescence. Co-infection of HIV with *M. tuberculosis* appears to expedite the downregulation of CD27 and CD28 compared to HIV infection alone. It is increasingly evident that chronic HIV infection hastens the onset of chronic immune activation, precipitating premature immunosenescence and heightening susceptibility to TB infection. Nevertheless, recent investigations propose that *M. tuberculosis* may also detrimentally impact HIV disease by augmenting viral transmission and facilitating HIV entry into immune cells.

This is difficult to overcome, as the formation of BCG booster vaccines is limited in that the type of vaccine function is short lived, would mostly be beneficial for the younger population, and may not necessarily prevent re-occurrence of infection because of *M. tuberculosis* and/or HIV induced immunosenescence. For most TB vaccine participants entering preclinical trials and clinical trials, their routine trials are limited. In most cases, they can be differentiated mostly by the magnitude of antigen-specific T-cell responses (Rodo et al., 2019). Studies should aim on finding promising protective antigens that are not confined only in inducing cellular immunity. The role of antibodies in TB has been controversial and should be taken into consideration in the architecture of TB vaccines (Li et al., 2017). In addition, adjuvants are mostly needed for vaccines to exert an abundant protective immune response against pathogens, which can enhance the vaccine efficacy for a continuous amount of time (Agger, 2016). In conclusion, new methods of technologies should be assessed in correlation with vaccines research and development. TB vaccine modules cannot be limited to its minute field and should be dealt with from the experiences of other successful vaccines such as Hib and meningococcal conjugate vaccines (Mascola and Fauci, 2020). In the last century, BCG vaccines have saved numerous lives worldwide. With the rapid changes of science and technologies, we believe that BCG vaccination methodologies and development as well as continuous efforts for a vaccine for HIV will be essential and vital in research pathway goals and will prove its positivity roles in public health and beyond.

## References

[B1] Agger E. M. (2016). Novel adjuvant formulations for delivery of anti-tuberculosis vaccine candidates. Adv. drug Deliv. Rev. 102, 73–82. 10.1016/j.addr.2015.11.012 26596558 PMC4870161

[B2] Appay V. Almeida J. R. Sauce D. Autran B. Papagno L. (2007). Accelerated immune senescence and HIV-1 infection. Exp. Gerontol. 42 (5), 432–437. 10.1016/j.exger.2006.12.003 17307327

[B3] Appay V. Dunbar P. R. Callan M. Klenerman P. Gillespie G. M. A. Papagno L. (2002). Memory CD8+ T cells vary in differentiation phenotype in different persistent virus infections. Nat. Med. 8 (4), 379–385. 10.1038/nm0402-379 11927944

[B4] Arbeláez M. P. Nelson K. E. Muñoz A. (2000). BCG vaccine effectiveness in preventing tuberculosis and its interaction with human immunodeficiency virus infection. Int. J. Epidemiol. 29 (6), 1085–1091. PMID: 11101552. 10.1093/ije/29.6.1085 11101552

[B5] Bobak C. A. Natarajan H. Gandhi T. Grimm S. L. Nishiguchi T. Koster K. (2022). Increased DNA methylation, cellular senescence and premature epigenetic aging in Guinea pigs and humans with tuberculosis. Aging (Albany NY) 14 (5), 2174–2193. 10.18632/aging.203936 35256539 PMC8954968

[B6] Bruchfeld J. Correia-Neves M. Kallenius G. (2015). Tuberculosis and HIV coinfection. Cold Spring Harb. Perspect. Med. 5 (7), a017871. 10.1101/cshperspect.a017871 25722472 PMC4484961

[B7] Chai Q. Lu Z. Liu C. H. (2020). Host defense mechanisms against *Mycobacterium tuberculosis* . Cell. Mol. life Sci. CMLS 77 (10), 1859–1878. 10.1007/s00018-019-03353-5 31720742 PMC11104961

[B8] Choremi-Papadopoulou H. Viglis V. Gargalianos P. Kordossis T. Iniotaki-Theodoraki A. Kosmidis J. (1994). Downregulation of CD28 surface antigen on CD4+ and CD8+ T lymphocytes during HIV-1 infection. J. Acquir Immune Defic. Syndr. (1988) 7 (3), 245–253. 7906302

[B9] Crooke S. N. Ovsyannikova I. G. Poland G. A. Kennedy R. B. (2019). Immunosenescence: a systems-level overview of immune cell biology and strategies for improving vaccine responses. Exp. Gerontol. 124, 110632. Epub 2019 Jun 13. PMID: 31201918; PMCID: PMC6849399. 10.1016/j.exger.2019.110632 31201918 PMC6849399

[B10] Cyktor J. C. Carruthers B. Stromberg P. Flaño E. Pircher H. Turner J. (2013). Killer cell lectin-like receptor G1 deficiency significantly enhances survival after mycobacterium tuberculosis infection. Infect. Immun. 81 (4), 1090–1099. 10.1128/iai.01199-12 23340310 PMC3639586

[B11] Deeks S. G. Verdin E. McCune J. M. (2012). Immunosenescence and HIV. Curr. Opin. Immunol. 24 (4), 501–506. 10.1016/j.coi.2012.05.004 22658763

[B12] Diedrich C. R. Flynn J. L. (2011). HIV-1/mycobacterium tuberculosis coinfection immunology: how does HIV-1 exacerbate tuberculosis? Infect. Immun. 79 (4), 1407–1417. 10.1128/IAI.01126-10 21245275 PMC3067569

[B13] Dock J. N. Effros R. B. (2011). Role of CD8 T cell replicative senescence in human aging and in HIV-mediated immunosenescence. Aging Dis. 2 (5), 382–397. 22308228 PMC3269814

[B14] Dockrell H. M. Smith S. G. (2017). What have we learnt about BCG vaccination in the last 20 Years? Front. Immunol. 8 (13 Sept), 1134. 10.3389/fimmu.2017.01134 28955344 PMC5601272

[B15] Effros R. B. (2007). Role of T lymphocyte replicative senescence in vaccine efficacy. Vaccine 25 (4), 599–604. 10.1016/j.vaccine.2006.08.032 17014937

[B16] Faurholt-Jepsen D. Range N. Praygod G. Jeremiah K. Faurholt-Jepsen M. Aabye M. G. (2013). BCG protects against tuberculosis irrespective of HIV status: a matched case-control study in Mwanza, Tanzania. Thorax 68 (3), 288–289. Epub 2012 Aug 24. PMID: 22923459. 10.1136/thoraxjnl-2012-201971 22923459

[B17] Gonzalez V. D. Falconer K. Blom K. G. Reichard O. Mørn B. Laursen A. L. (2009). High levels of chronic immune activation in the T-cell compartments of patients coinfected with hepatitis C virus and human immunodeficiency virus type 1 and on highly active antiretroviral therapy are reverted by alpha interferon and ribavirin treatment. J. Virol. 83 (21), 11407–11411. 10.1128/JVI.01211-09 19710147 PMC2772767

[B18] Gui J. Mustachio L. M. Su D. M. Craig R. W. (2012). Thymus size and age-related thymic involution: early programming, sexual dimorphism, progenitors and stroma. Aging Dis. 3 (3), 280–290. 10.14336/AD.2012.0300280 22724086 PMC3375084

[B19] Gupta A. Wood R. Kaplan R. Bekker L. G. Lawn S. D. (2012). Tuberculosis incidence rates during 8 years of follow-up of an antiretroviral treatment cohort in South Africa: comparison with rates in the community. PLoS One 7 (3), e34156. 10.1371/journal.pone.0034156 22479548 PMC3316623

[B20] Hamann D. Roos M. T. van Lier R. A. (1999). Faces and phases of human CD8 T-cell development. Immunol. Today 20 (4), 177–180. 10.1016/s0167-5699(99)01444-9 10203715

[B21] Hazenberg M. D. Otto S. A. van Benthem B. H. B. Roos M. T. L. Coutinho R. A. Lange J. M. A. (2003). Persistent immune activation in HIV-1 infection is associated with progression to AIDS. AIDS 17 (13), 1881–1888. 10.1097/00002030-200309050-00006 12960820

[B22] Hunt P. W. Brenchley J. Sinclair E. McCune J. M. Roland M. Page-Shafer K. (2008). Relationship between T cell activation and CD4+ T cell count in HIV-seropositive individuals with undetectable plasma HIV RNA levels in the absence of therapy. J. Infect. Dis. 197 (1), 126–133. 10.1086/524143 18171295 PMC3466592

[B23] Kaufmann S. H. Dorhoi A. (2013). Inflammation in tuberculosis: interactions, imbalances and interventions. Curr. Opin. Immunol. 25 (4), 441–449. 10.1016/j.coi.2013.05.005 23725875

[B24] Kovaiou R. D. Weiskirchner I. Keller M. Pfister G. Cioca D. P. Grubeck-Loebenstein B. (2005). Age-related differences in phenotype and function of CD4+ T cells are due to a phenotypic shift from naive to memory effector CD4+ T cells. Int. Immunol. 17 (10), 1359–1366. 10.1093/intimm/dxh314 16141244

[B25] Kushner E. J. Weil B. R. MacEneaney O. J. Morgan R. G. Mestek M. L. Van Guilder G. P. (2010). Human aging and CD31+ T-cell number, migration, apoptotic susceptibility, and telomere length. J. Appl. Physiol. (1985) 109 (6), 1756–1761. 10.1152/japplphysiol.00601.2010 20864561 PMC3006402

[B26] Kwan C. K. Ernst J. D. (2011). HIV and tuberculosis: a deadly human syndemic. Clin. Microbiol. Rev. 24 (2), 351–376. 10.1128/CMR.00042-10 21482729 PMC3122491

[B27] Larson E. C. Ellis-Connell A. L. Rodgers M. A. Gubernat A. K. Gleim J. L. Moriarty R. V. (2023). Intravenous Bacille calmette–guérin vaccination protects simian immunodeficiency virus-infected macaques from tuberculosis. Nat. Microbiol. 8, 2080–2092. 10.1038/s41564-023-01503-x 37814073 PMC10627825

[B28] Lee S. A. Sinclair E. Hatano H. Hsue P. Y. Epling L. Hecht F. M. (2014). Impact of HIV on CD8+ T cell CD57 expression is distinct from that of CMV and aging. PLoS One 9 (2), e89444. 10.1371/journal.pone.0089444 24586783 PMC3937334

[B29] Li H. Wang X. X. Wang B. Fu L. Liu G. Lu Y. (2017). Latently and uninfected healthcare workers exposed to TB make protective antibodies against *Mycobacterium tuberculosis* . Proc. Natl. Acad. Sci. U. S. A. 114 (19), 5023–5028. 10.1073/pnas.1611776114 28438994 PMC5441709

[B30] Luca S. Mihaescu T. (2013). History of BCG vaccine. Maedica 8 (1), 53–58. Available at: www.ncbi.nlm.nih.gov/pmc/articles/PMC3749764/ (Accessed January 16, 2024). 24023600 PMC3749764

[B31] Mascola J. R. Fauci A. S. (2020). Novel vaccine technologies for the 21st century. Nat. Rev. Immunol. 20 (2), 87–88. 10.1038/s41577-019-0243-3 31712767 PMC7222935

[B32] Meissner E. G. Duus K. M. Loomis R. D'Agostin R. Su L. (2003). HIV-1 replication and pathogenesis in the human thymus. Curr. HIV Res. 1 (3), 275–285. 10.2174/1570162033485258 15046252 PMC4425345

[B33] Mojumdar K. Vajpayee M. Chauhan N. K. Singh A. Singh R. Kurapati S. (2012). Altered T cell differentiation associated with loss of CD27 and CD28 in HIV infected Indian individuals. Cytom. B Clin. Cytom. 82 (1), 43–53. 10.1002/cyto.b.20610 21695776

[B34] Norouzi S. Aghamohammadi A. Mamishi S. Rosenzweig S. D. Rezaei N. (2012). Bacillus Calmette-Guérin (BCG) complications associated with primary immunodeficiency diseases. J. Infect. 64 (6), 543–554. 10.1016/j.jinf.2012.03.012 22430715 PMC4792288

[B35] Okamura T. Shimizu Y. Asaka M. N. Kanuma T. Tsujimura Y. Yamamoto T. (2021). Long-term protective immunity induced by an adjuvant-containing live-attenuated AIDS virus. NPJ Vaccines 6 (1), 124. PMID: 34686680; PMCID: PMC8536741. 10.1038/s41541-021-00386-5 34686680 PMC8536741

[B36] Olmo-Fontánez A. M. Turner J. (2022). Tuberculosis in an aging world. Pathogens 11 (10), 1101. 10.3390/pathogens11101101 36297158 PMC9611089

[B37] Papagno L. Spina C. A. Marchant A. Salio M. Rufer N. Little S. (2004). Immune activation and CD8+ T-cell differentiation towards senescence in HIV-1 infection. PLoS Biol. 2 (2), E20. 10.1371/journal.pbio.0020020 14966528 PMC340937

[B38] Qu M. Zhou X. Li H. (2021). BCG vaccination strategies against tuberculosis: updates and perspectives. Hum. vaccines Immunother. 17 (12), 5284–5295. 10.1080/21645515.2021.2007711 PMC8903987 34856853

[B39] Reyes A. Ortiz G. Duarte L. F. Fernández C. Hernández-Armengol R. Palacios P. A. (2023). Contribution of viral and bacterial infections to senescence and immunosenescence. Front. Cell. Infect. Microbiol. 13, 1229098. 10.3389/fcimb.2023.1229098 37753486 PMC10518457

[B40] Rodo M. J. Rozot V. Nemes E. Dintwe O. Hatherill M. Little F. (2019). A comparison of antigen-specific T cell responses induced by six novel tuberculosis vaccine candidates. PLoS Pathog. 15 (3), e1007643. 10.1371/journal.ppat.1007643 30830940 PMC6417742

[B41] Rodriguez B. Sethi A. K. Cheruvu V. K. Mackay W. Bosch R. J. Kitahata M. (2006). Predictive value of plasma HIV RNA level on rate of CD4 T-cell decline in untreated HIV infection. JAMA 296 (12), 1498–1506. 10.1001/jama.296.12.1498 17003398

[B42] Sauce D. Larsen M. Fastenackels S. Duperrier A. Keller M. Grubeck-Loebenstein B. (2009). Evidence of premature immune aging in patients thymectomized during early childhood. J. Clin. Investigation 119 (10), 3070–3078. 10.1172/jci39269 PMC2752077 19770514

[B43] Shankar E. M. Velu V. Kamarulzaman A. Larsson M. (2015). Mechanistic insights on immunosenescence and chronic immune activation in HIV-tuberculosis co-infection. World J. Virology 4 (1), 17–24. 10.5501/wjv.v4.i1.17 25674514 PMC4308524

[B44] Shankar E. M. Vignesh R. Ellegård R. Barathan M. Chong Y. K. Bador M. K. (2014). HIV-Mycobacterium tuberculosis co-infection: a 'danger-couple model' of disease pathogenesis. Pathog. Dis. 70 (2), 110–118. 10.1111/2049-632X.12108 24214523

[B45] Sharp M. Dohme L. L. C. (2023). TICE BCG (merck Sharp and Dohme LLC): FDA package insert. MedLibrary.Org. Available at: www.medlibrary.org/lib/rx/meds/tice-bcg/ (Accessed January 16, 2024).

[B46] Silvestri G. Sodora D. L. Koup R. A. Paiardini M. O'Neil S. P. McClure H. M. (2003). Nonpathogenic SIV infection of sooty mangabeys is characterized by limited bystander immunopathology despite chronic high-level viremia. Immunity 18 (3), 441–452. 10.1016/s1074-7613(03)00060-8 12648460

[B48] Sokoya T. Steel H. C. Nieuwoudt M. Rossouw T. M. (2017). HIV as a cause of immune activation and immunosenescence. Mediat. Inflamm. 2017, 6825493. 10.1155/2017/6825493 PMC5676471 29209103

[B49] Stahl E. C. Brown B. N. (2015). Cell therapy strategies to combat immunosenescence. Organogenesis 11 (4), 159–172. 10.1080/15476278.2015.1120046 26588595 PMC4879890

[B50] Tanner R. Villarreal-Ramos B. Vordermeier H. M. McShane H. (2019). The humoral immune response to BCG vaccination. Front. Immunol. 10, 1317. 10.3389/fimmu.2019.01317 31244856 PMC6579862

[B51] Trickey A. Zhang L. Sabin C. A. Sterne J. A. C. (2022). Life expectancy of people with HIV on long-term antiretroviral therapy in Europe and North America: a cohort study. Lancet Healthy Longev. 3, S2. 10.1016/S2666-7568(22)00063-0

[B52] Walker N. F. Meintjes G. Wilkinson R. J. (2013). HIV-1 and the immune response to TB. Future Virol. 8 (1), 57–80. 10.2217/fvl.12.123 23653664 PMC3644063

[B53] Ye P. Kirschner D. E. Kourtis A. P. (2004). The thymus during HIV disease: role in pathogenesis and in immune recovery. Curr. HIV Res. 2 (2), 177–183. 10.2174/1570162043484898 15078181

[B54] Zwerling A. Behr M. A. Verma A. Brewer T. F. Menzies D. Pai M. (2011). The BCG World Atlas: a database of global BCG vaccination policies and practices. PLoS Med. 8 (3), e1001012. Epub 2011 Mar 22. PMID: 21445325; PMCID: PMC3062527. 10.1371/journal.pmed.1001012 21445325 PMC3062527

